# A Case of Hernia Congenita

**Published:** 1803-07-01

**Authors:** W. Robertson

**Affiliations:** One of the Surgeons to the Kelso Dispensary


### *A* Case *of* Hernia Congenita, *by* Mr. W. Robertson, *One of the Surgeons to the Kelso Dispensary.*

JOHN TURNBULL, æt. 17, of a delicate habit of body, a taylor by trade, had been occasionally subject to a scrotal hernia from his infancy to the age of twelve years, when using any uncommon exertion; but by the application of a bandage properly adapted to the part, it entirely disappeared for nearly the space of five years. On the 18th of September, 1802, being the harvest time, he ate a great quantity of green peas, which disagreed with his stomach, brought on a violent reaching and vomiting; in consequence of which exertion, his complaint returned in a very violent form, attended with much pain in the part. He continued in that state till the 20th of the same month, when the assistance of one of the medical faculty was requested, who blooded him freely, and used other means for its reduction, without effect. On the 22d, I was desired to visit him, and found him in great distress, complaining of great pain in the part, a constant reaching and vomiting of every thing he received into his stomach, with a total obstruction in his bowels; his pulse was full and strong. I blooded him almost ad deliquium animi, immediately after raised his feet over a person's shoulder that was standing, and caused his body to be a good deal agitated in that state, and afterwards attempted the reduction of the hernia, but without success. He was then ordered to lay constantly with his feet and breech raised higher than his head, and to keep linen rags, doubled three or four times, constantly wet, with a cold saturnine lotion at the part; a position and application which I have seen in several instances attended with happy effects. He was likewise ordered to have an injection of warm water, with soap dissolved in it; and after its operation was over, to have injections of tobacco smoke alternated with one of warm water and laudanum frequently repeated, at the same time to take a smart dose of calomel and jalap, which his stomach rejected; the dose was administered a second time with no better success. I visited him on the 23d, found all applications ordered had failed: He was then put into the warm bath for a quarter of an hour. After coming out of it, the reduction was again attempted without being able to make any alteration in the part, which was now become exceedingly painful. When handled, there was likewise a great degree of pain and tension over the whole abdomen. His situation now became very alarming. On this I held a consultation with Dr. Wilson, of this place, and Messrs. Bruice and Jack, surgeons. Having taken his case into consideration, and found that all the means that are generally applied with advantage in similar complaints, had been used in vain, we were unanimously of opinion, that an operation was the only chance which remained to rescue him from death, which seemed to be fast approaching.

The danger of his situation being explained to himself and his friends, an operation was proposed as the only resource left, which he agreed to.

He was laid in a proper position, having previously prepared what was necessary for the occasion.

I began the incision about half an inch above the top of the tumour with a scalpel, and carried it nearly to the bottom of the scrotum, the extent which the rupture occupied. The cellular substance and the vaginal coat were laid open, by raising upon the directory the fibres by parcels, and dividing them with a pair of sharp scissars, which I found a preferable instrument to the probe pointed bistoury commonly used, both for safety and expedition. Having cautiously scratched a hole in the hernial sac, there issued out about six ounces of a sanious coloured fluid; the opening being enlarged, the testicle was exposed to view. In order to prevent it from falling out of the scrotum, and to exclude the air, I was under the necessity of stitching the under part of the incision, which secured the testicle compleatly till the rest of the operation was finished. The sac was then laid open its whole length. The contents consisted of a large portion of the omentum of a livid black colour. The portion of gut was about the size of a walnut, and of the colour of a tamirand stone; though of a very bad appearance, it was thought advisable to reduce them, with an idea that they might recover their colour after being returned into their natural situation.

The protruded parts had acquired such a size by the inflamed state they were in, that it was found impracticable to effect the reduction without enlarging the opening at the ring, which was done with the scissars, directed by the fore finger. After the reduction was accomplished, and the lips of the wound drawn close together by adhesive straps, and other proper dressings applied and secured by the T bandage, he was carried to bed and laid in a proper posture. He was ordered a little wine negus, and an emollient injection, which procured a plentiful evacuation from his his bowels. Upon visiting him two hours afterwards, his pulse was become quite hard and full. He was then blooded to the extent of twelve ounces. The blood was sizy. He was ordered a cooling cathartic, composed of salts, senna, and tamarinds, which operated freely, and gave him great relief. After this, matters seemed to go on pretty well. The wound was dressed on the 26th, looked well, and almost healed by the first intention.

On the 27th, he was seized with violent shooting pains, with an increase of the tension over the whole abdomen, with a constant vomiting of every thing he took into his stomach, and with a total obstruction of his bowels, so that no injection would pass. He became quite restless, and his pulse more full and frequent. He was blooded again in the arm; but lest too much blood might be abstracted from the general mass, a great number of leeches was applied over the whole of the abdomen, which was also to be frequently fomented with emollient decoctions. The pain still continued. A large blister was applied, which rose well, but the abdomen still continued to increase in size. The total obstruction of his bowels and vomiting continued the same. His countenance became ghastly, his pulse hectic and feeble; nothing but death was daily expected. Mr. Jack, who lived in the neighbourhood, and visited him daily, when dressing the wound, observed a large tumour pointing at the part where the incision was made for the operation; on this, he introduced a trocar and canula and drew off nearly four pounds of matter. The swelling of the abdomen diminished considerably. He had on the same day, got nine grains of calomel made into two pills, with two grains of the extract of opium, which operated powerfully after the evacuation of the matter. For a long time afterwards, he was troubled with a colloquative diarrhœa. The stools being of a very dark colour, and having a fœtid smell, for which he had a few gentle doses of rhubarb with an opiate at bed time. The wound continued to discharge a good deal of matter daily. In the course of the discharge there appeared at the wound a decayed substance, which was laid hold of and pulled out. It proved to be a portion of the omentum, measuring about eight inches in length and two in breadth, of an irregular shape. In some subsequent dressings, two smaller portions came out. He was ordered a nourishing diet, consisting of rich broths and a portion of wine daily. After the diarrhœa left him, he was put upon a course of the decoction of the bark acidulated with the sp. vitriol. dilut.

D 2 The

The hectic fever began to abate, the wound healed up, and he recovered his strength daily, and is now quite healthy and following his employment.

Is it not reasonable to suppose, that in the above case, from the great distension of the abdomen, that the omentum was in a high state of inflammation before the operation was performed, and that by the vigorous prosecution of the antiphlogistic plan, the viscera concerned in the disease were brought into a state of suppuration, and prevented from running into gangrene, and that by these means the life of the patient was saved?

Would not the same termination have taken place, allowing that the diseased portion of the omentum which was included in the sac, had been cut off?

The whole of the matter that was evacuated at different times, was calculated at five pounds. Is it not likely that this great quantity of matter pressing by its weight upon the bowels, was the cause of the vomiting and of the total obstruction of the bowels? It may appear a little surprizing why I performed most of the operation with the scissars, an instrument never mentioned by writers on surgery as a suitable one for that purpose; but whoever tries will find them a very convenient one, as they cut the parcels of fibres raised upon the directory much more readily than the bistoury commonly in use, and they are certainly safer and more expeditious in the hands of those who have not had much practice in this operation.

